# Nanocurcumin in Oral Squamous Cancer Cells and Its Efficacy as a Chemo-Adjuvant

**DOI:** 10.7759/cureus.24678

**Published:** 2022-05-02

**Authors:** Diptasree Mukherjee, Prakruti Dash, Balamurugan Ramadass, Manaswini Mangaraj

**Affiliations:** 1 Biochemistry, All India Institute of Medical Sciences, Bhubaneshwar, IND; 2 Biochemistry, All India Institute of Medical Sciences, Bhubaneswar, IND

**Keywords:** curcumin, cytotoxicity, adjuvant therapy, cetuximab, nanocurcumin, oral cancer

## Abstract

Oral squamous cell carcinoma is the sixth most common cancer worldwide. Despite the available treatment, the survival rate is poor. The addition of agents to make chemotherapeutics safer and more effective is important. Curcumin is a common Indian spice that has shown anticarcinogenic properties. It has been possible to overcome its poor bio-availability using nanotechnology. We aimed to investigate the adjuvant effect of nanocurcumin (NC ~ 200 nm size) treatment on cetuximab (epidermal growth factor receptor inhibitor) in oral squamous cancer cells (KB 3-1 cell). Cancer cells were cultured and treated for 24 hours with cetuximab and NC, in various doses to find the drugs' half-maximal inhibitory concentration (IC_50_). Experiments were conducted with a combination dose of both and sensitization treatment with NC before cetuximab with cytotoxicity assessment by 3-(4,5-dimethylthiazol-2-yl)-2,5-diphenyltetrazolium bromide assay. One-way analysis of variance (ANOVA) was used to compare different treatment groups. We found a concentration-dependent cancer cell death with NC, which was significant compared to cetuximab (p <0.001). The combination treatment group had highly significant cell death (p <0.0001) compared to a single drug, and the NC sensitization caused substantial cell death compared to a single cetuximab treatment (p<0.01). Our study findings indicate the potential chemo-adjuvant effect of NC in oral cancer.

## Introduction

Oral squamous cell carcinoma (OSCC) is the sixth most common cancer. It constitutes 5% of all malignant tumors globally and 45% of all malignant tumors in India, with less than 50% five-year survival [1]. In the Globocan report of 2020, for India, lip and oral cavity cancer incidence is 10.3% [2]. The development of OSCC has been attributed to multiple risk factors like tobacco use, alcohol consumption, infections with agents like Epstein-Barr virus, and Human papillomavirus, which are highly prevalent in India [3]. The treatment for OSCC requires a multi-modal therapeutic approach with surgery, radiotherapy, and with or without chemotherapy [4]. Cetuximab, an epidermal growth factor receptor (EGFR) monoclonal antibody, is a form of targeted therapy, that has increased treatment efficacy and improved overall survival but has shown significant adverse effects and development of drug resistance [5]. Hence use of combination therapy to make chemotherapeutics more effective is the need of the hour.

Curcumin is a phytochemical isolated from the turmeric plant (Curcuma longa). It has been reported to have multiple therapeutic properties such as antiviral, anti-inflammatory, antioxidant, antitumor, antimicrobial, cardioprotective, anti-arthritic, chemopreventive, and anticarcinogenic properties [6]. Studies have shown the therapeutic role of curcumin in various cancers, including oral cancer. Curcumin acts on numerous molecular targets like signal transducer and activator of transcription 3 (STAT3), activator protein 1 (AP-1), protein kinase B (PKB), notch homolog 1 translocation-associated (Drosophila) (Notch1), nuclear factor κβ (NF-κβ), wingless and int1 (Wnt) and mitogen-activated protein kinase (MAPK) [6]. Epidermal growth factor receptor and its downstream signalling pathways play a key role in oral squamous carcinoma pathogenesis and are inhibited by curcumin [7]. Despite its benefits, multiple factors often limit the practical application of curcumin, such as physicochemical instability, rapid metabolism, low pharmacokinetics, and bioavailability. Nevertheless, these barriers are solved by nanotechnology and using nanoformulations of curcumin [8]. Different approaches to curcumin can improve its physicochemical characteristics and enable its efficient use. For that purpose, formulations including liposomes, nanoparticles, micelles, and phospholipid complexes have been described in the reference sources [9]. In epithelial-type cancers, nanocurcumin formulations have shown favorable results adding to the evidence of its therapeutic role in cancer treatment as an adjuvant [10,11]. This study aimed to assess the adjuvant effects of nanoparticle nanocurcumin (NC) on cetuximab treatment in OSCC cells.

This article was previously presented as an abstract at the 47^th ^ National Conference of Association of Clinical Biochemists of India (ACBICON) held from 12th to 15th December 2021.

## Materials and methods

This study to evaluate the efficacy of nanocurcumin (NC) as a chemo-adjuvant for oral cancer was approved by the Institutional Ethics Committee of All India Institute of Medical Sciences (AIIMS), Bhubaneswar, India (approval No. IEC/AIIMS BBSR/PG THESIS/2018-19/15).

Cell culture

Human oral cancer cells (KB 3-1 cell) from National Centre for Cell Science (NCCS), Pune, India, were cultured in T25 flasks. Complete media (CDMEM) used for cell culture was prepared with Dulbecco's Modified Eagle Medium (containing 4.5gms glucose/litre, sodium bicarbonate, and sodium pyruvate with L-glutamine) along with 10% fetal bovine serum and 1% antibiotic antimycotic solution (10,000U penicillin, 10mg streptomycin, 25 µg amphotericin B per ml in 0.9% saline). Cells were incubated at 37°C with 5% CO_2_, and 95% humidified air in the CO_2_ incubator. All the experiments were done in triplicates, and each independent experiment was repeated thrice.

MTT assay

The live cells with an active metabolism, when treated with 3-(4,5-dimethylthiazol-2-yl-2),5-diphenyltetrazolium bromide (MTT), convert MTT into a purple-colored formazan crystal with an absorbance of 570nm. On dying, the cells lose their ability to convert MTT into formazan, and hence color formation functions as a convenient marker of only the viable cells. The mechanism of MTT reduction into formazan likely involves a reaction with nicotinamide adenine dinucleotide hydride (NADH) or similar, reducing molecules that transfer electrons to MTT [12].

A stock solution of MTT reagent was prepared of 5mg/mL using reagents from the kit. The stock was syringe filtered and kept in a (-)20°C fridge for further use. For cell viability assay, 0.5mg/mL MTT reagent was prepared fresh every time from the stock solution with CDMEM. After treatment, media was changed from all wells, including control wells, and 0.5mg/mL of 100µL MTT reagent was added to all the wells in the 96 well plates. The plate was kept in the dark inside the CO_2 _incubator for two hours and then observed under a phase-contrast microscope (10x). The 96 well plates were taken out and placed in a horizontal shaker for about 10 minutes before taking a reading in the i-control microplate reader software (Tecan, Switzerland).

According to the MTT cell assay kit, specific absorbance (Abs) = [Abs (570nm) test - Abs (570nm) blank] - Abs (630nm) test

Cell viability was calculated as: Cell viability (%) = [O.D.(test)−O.D.(blank) / O.D.(control)−O.D.(blank)] × 100

(O.D. = optical density)

Treatment with cetuximab

A 2mg/ml cetuximab stock solution was prepared with 0.9% saline. Sequential doses of cetuximab were prepared from the stock solution (250, 500, 750, and 1000µg/mL) with CDMEM each time before treatment of the cells. Cells were harvested at 70% confluency from the T25 flasks, counted using a hemocytometer, and plated as 100µL of 1x10^4^ cells/well in flat bottomed 96 well plates in triplicates. The plated cells were incubated overnight in the CO_2_ incubator. The following day media was removed, and they were treated with serial doses of cetuximab of 100µL volume to each well. In the control wells, only media was changed. After 24 hours, cells were treated with MTT reagent following the manufacturer's guidelines as stated above, and absorbance was measured using an i-control microplate reader machine at wavelength 570nm with reference wavelength 630nm. The half-maximal inhibitory concentration (IC_50_) of cetuximab was calculated.

Treatment with NC

Nanocurcumin stock solution of 50mM concentration was prepared by dissolving in 0.5M sodium hydroxide and an immediate dilution in phosphate-buffered saline (1X). The stock's serial doses of NC were prepared in CDMEM of 5, 25, 50, 75, and 100µM. Like the cetuximab treatment group, cancer cells were plated, and the following day they were treated with serial doses of NC. After 24 hours of treatment, an MTT assay was performed, following which IC_50_ of NC was calculated.

Combined treatment with cetuximab and NC

The KB 3-1 cells were plated and the following day treated with IC_50_ dose of cetuximab, NC separately, and a combination of NC and cetuximab. After 24 hours of treatment, absorbance was measured following treatment with MTT reagent similarly as above.

Sensitized by NC before cetuximab

The KB 3-1 cells were plated and cultured for the subsequent experiments. One group was pre-treated with NC (at IC_50_ dose) overnight and the following 24 hours with cetuximab (at IC_50_ dose). Another group was treated only with 24 hours of cetuximab (IC_50_ dose). After drug treatment, absorbance was measured in the same way.

Statistical analysis

Categorical variables were summarized as frequencies (percentages). Tukey's post-hoc tests were used to compare categorical variables. One-way ANOVA was used to compare different treatment groups. The results are presented as mean with standard deviation (SD). The IC_50_ was calculated using a non-linear regression model. All tests were two-tailed, and statistical significance was determined if the p-value was <0.05. All statistical analyses were performed with GraphPad Prism 8.4.2 software (GraphPad Software Inc., San Diego, CA, USA).

## Results

**Figure 1:**
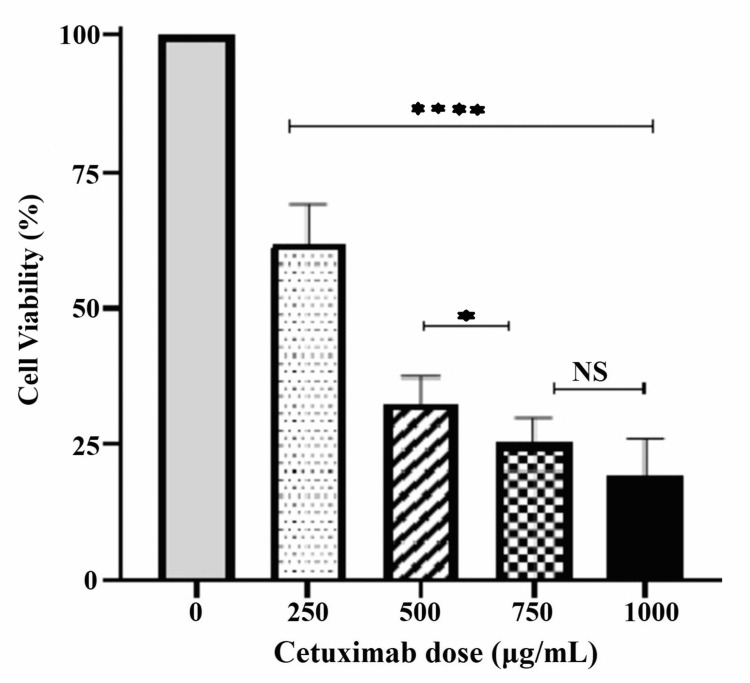
Cytotoxic effect of 24-hour cetuximab treatment The graph shows the cytotoxic effect of cetuximab on KB cell lines after 24 hours of treatment. All doses were significant compared to the control (p<0.0001). From the dose of 750µg/mL onwards the drug cytotoxicity plateaued as there was no significant cell death between the doses of 750µg/mL and 1000µg/mL. NS: Not significant

Our study found that treatment with sequential doses of cetuximab for 24 hours had cytotoxic effects on KB 3-1 cells in a dose-dependent manner. The cytotoxicity for the doses 250μg/mL, 500μg/mL, 750μg/mL and 1000μg/mL was 38.2%, 67.5%, 74.8% and 80.7%, respectively (Figure 1).

**Figure 2:**
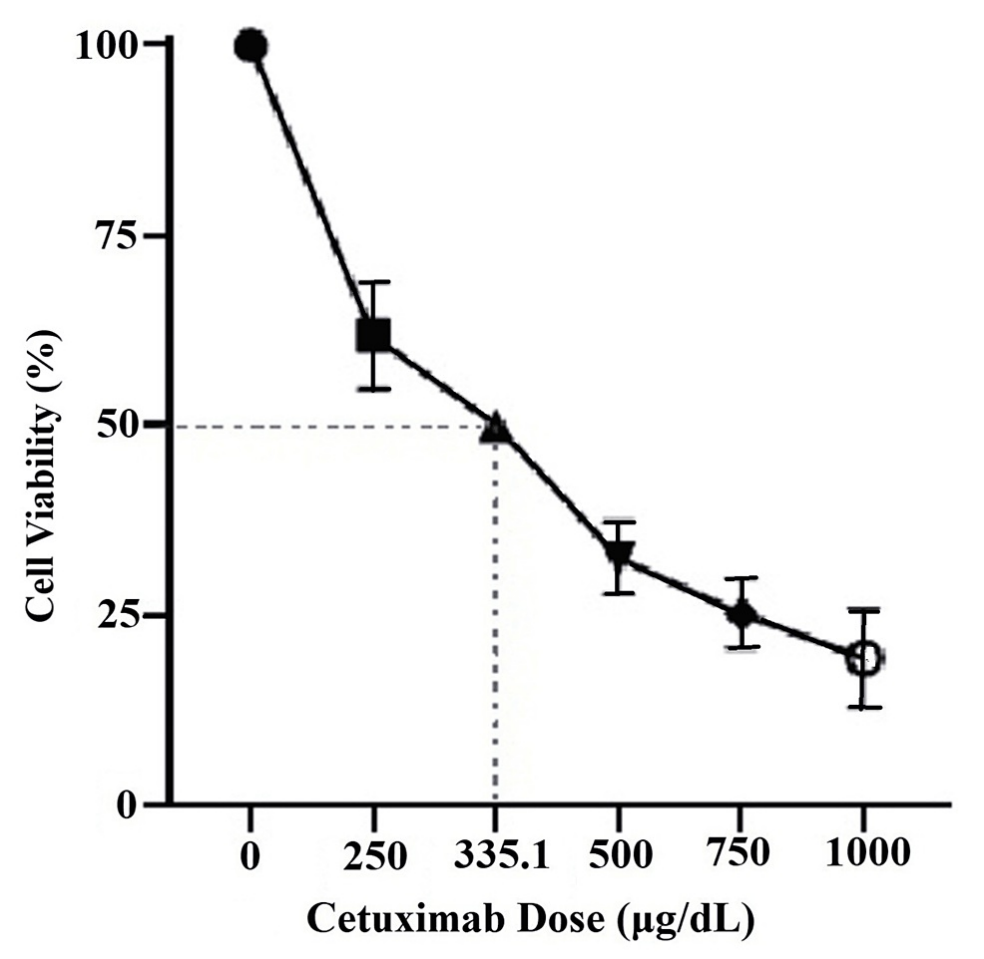
Dose-response curve of 24-hour cetuximab treatment The graph shows the cell viability (%) of the KB cells treated with cetuximab for 24 hours. There was a decline in cell viability observed in a dose-dependent manner. Drug's IC_50_ was calculated using GraphPad Prism software and plotted on the dose-response curve to be 335.1µg/mL.

The cytotoxicity observed was significant at all the sequential doses compared to control (p <0.0001), with a plateauing effect after 750µg/mL. The IC_50_ of cetuximab was calculated to be 335.1µg/mL (Figure 2). For further experiments we used 330µg/mL as cetuximab IC_50._

**Figure 3:**
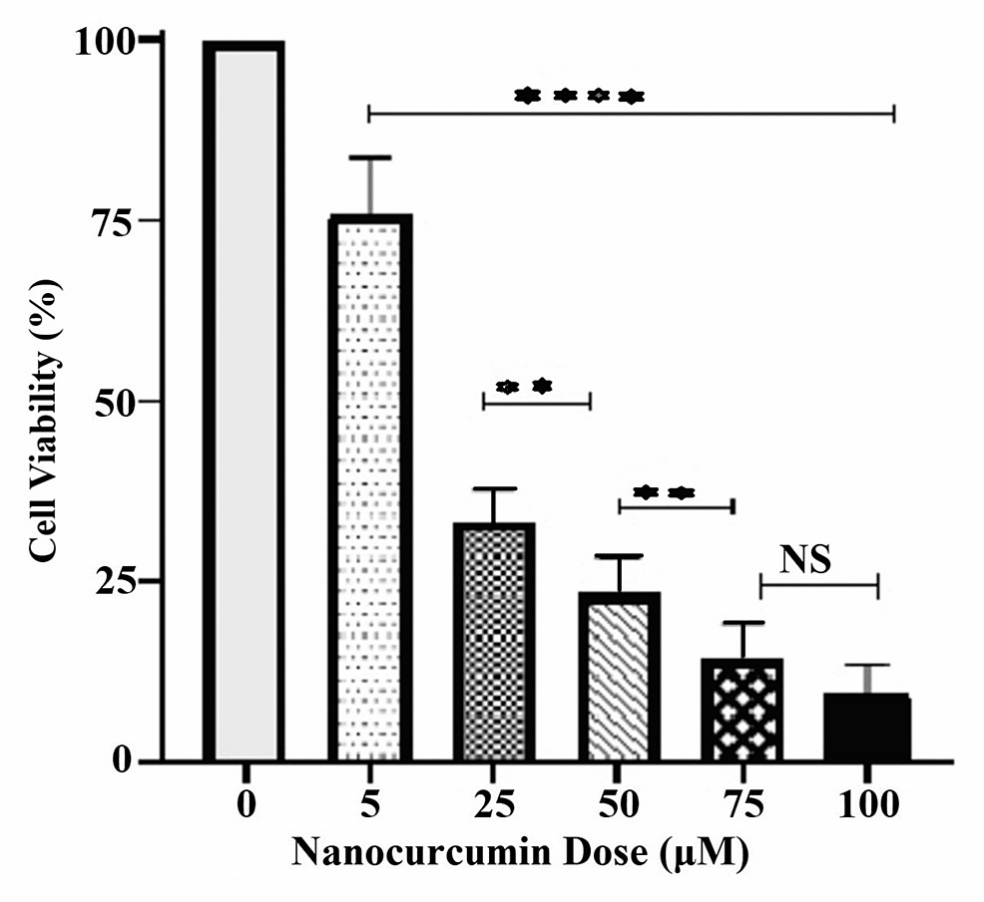
Cytotoxic effect of 24-hour nanocurcumin treatment The graph shows the cytotoxic effect of nanocurcumin (NC) on KB cells lines with 24 hours treatment. All doses were significant compared to control (p<0.0001). From the dose 75µM onwards the drug cytotoxicity plateaued as there was no significant cell death between the doses 75µM and 100µM. NS: Not significant

Similar dose-dependent cytotoxic effects were seen with sequential doses of NC. The cytotoxicity for the doses 5μM, 25μM, 50μM, 75μM and 100μM was 24.0%, 76.8%, 85.9% and 90.7%, respectively (Figure 3).

**Figure 4:**
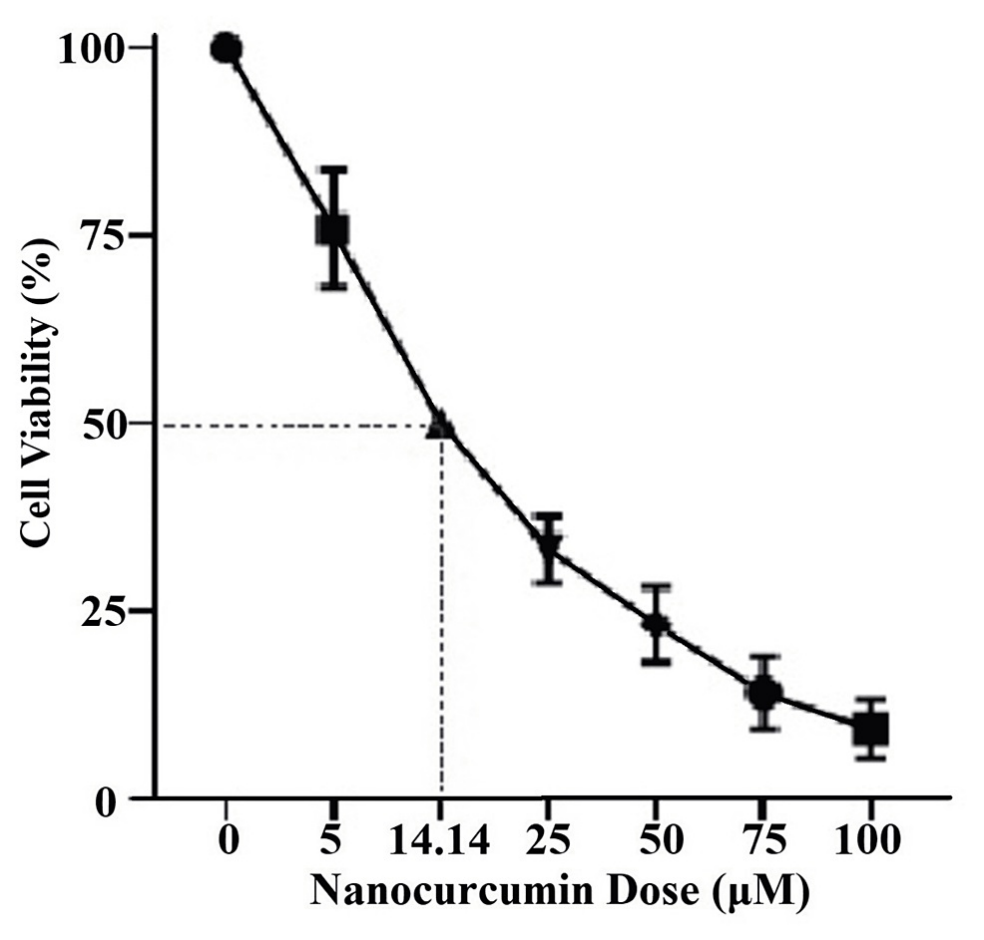
Dose-response curve of 24-hour nanocurcumin treatment The graph shows the cell viability (%) of the KB cells treated with nanocurcumin for 24 hours. There was a decline in cell viability in a dose-dependent manner. The IC_50_ of nanocurcumin was calculated using GraphPad Prism software and plotted on the dose-response curve to be14.14µM.

The cytotoxicity was significant at all doses. The IC_50_ of NC was calculated to be 14.14µM (Figure 4). For further experiments we used 15µM as NC IC_50_."

**Figure 5:**
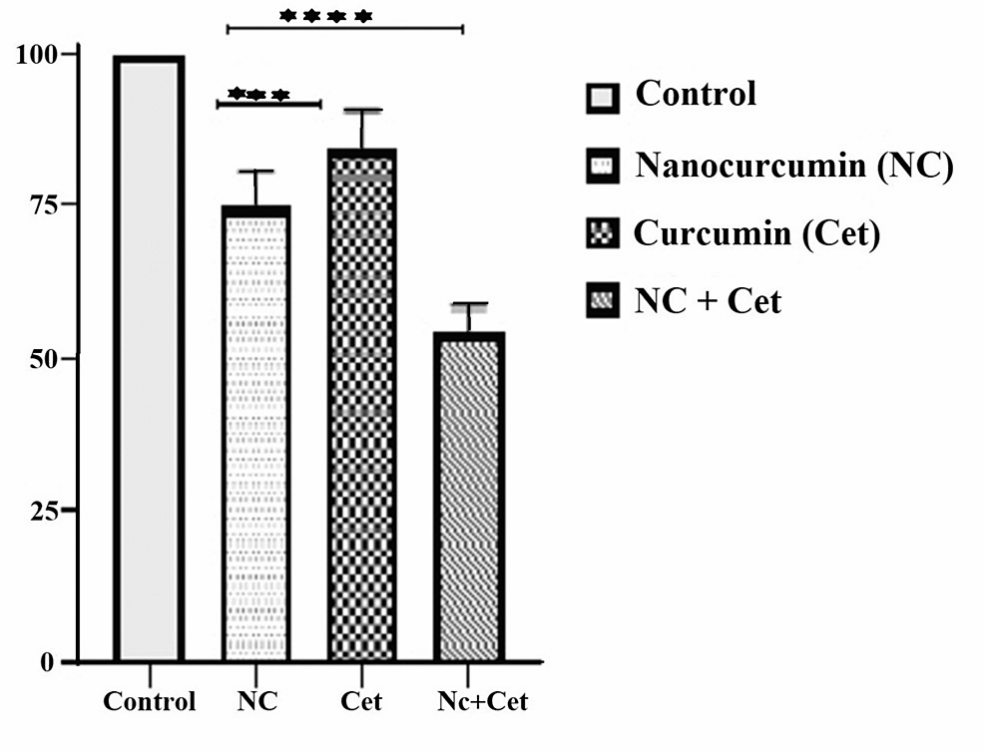
Cytotoxic effect of combined treatment with nanocurcumin (NC) and cetuximab The graph shows the cell viability (%) of the KB cells treated with the combination of IC_50_ doses of NC and cetuximab against a single treatment with NC and cetuximab for 24 hours. There was highly significant cell inhibition in the combined treatment group compared to the single treatment groups (p<0.0001).  The NC caused more cell death compared to cetuximab alone (p<0.001).

In the combination treatment experiments, we found the cytotoxicity of the NC alone, cetuximab alone, and their combination treatment to be 25.4%, 15.5%, and 46.0%, respectively (Figure 5).

The combination group had highly significant cell death (p <0.0001) compared to the single-drug treatments. Cell death by NC treatment was significant (p <0.001) against cetuximab treatment.

**Figure 6:**
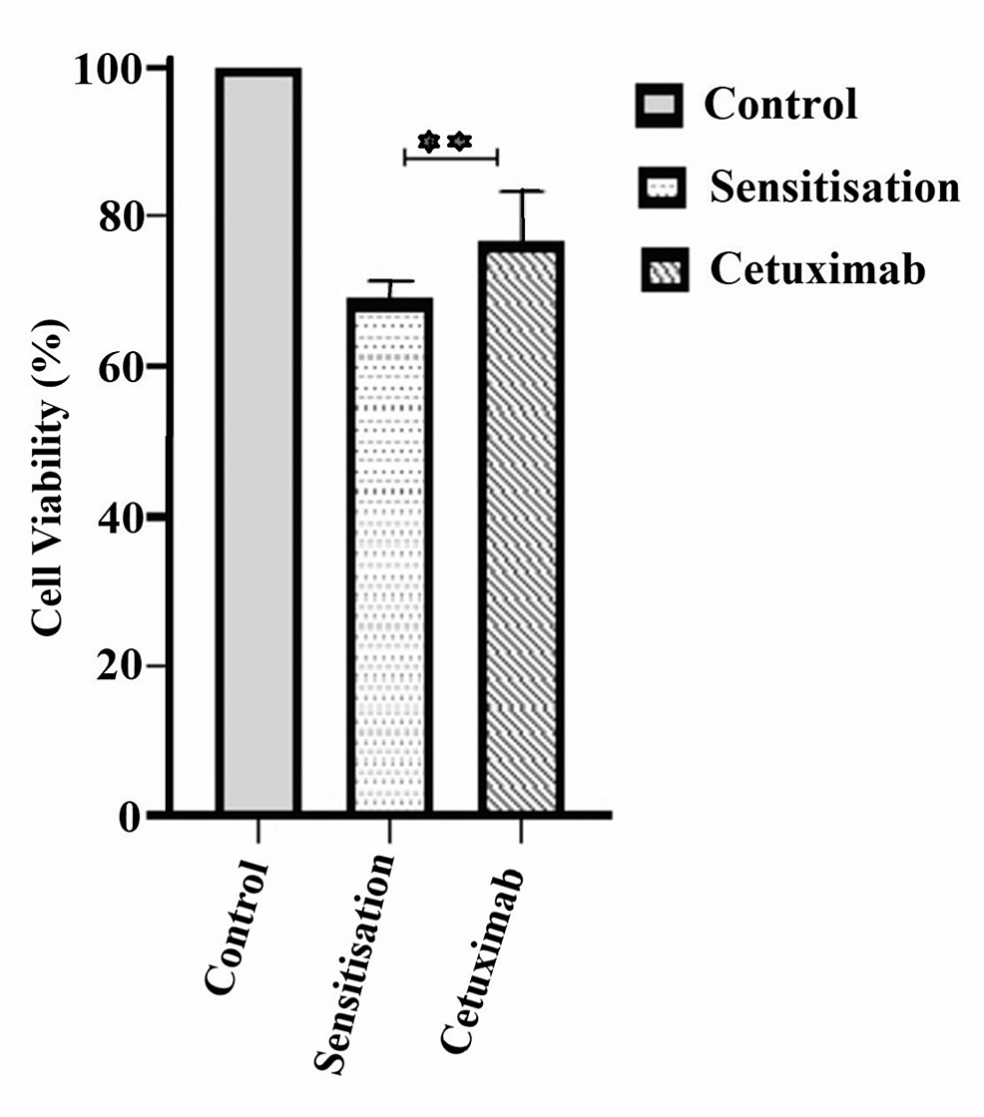
Cytotoxic effect of nanocurcumin sensitised cetuximab against cetuximab alone treatment The graph shows the cell viability (%) of the KB cells sensitised with nanocurcumin prior to 24 hours of cetuximab treatment. There was significant cell growth inhibition in the sensitised treatment group compared to the cetuximab single treatment group (p<0.01).

Oral cancer cells sensitized with NC before cetuximab treatment showed a cytotoxic effect of 30.9%. The results of this experiment were compared to the single treatment effect of cetuximab and the combined therapy of NC-cetuximab. Sensitization to NC caused significant cell death compared to cetuximab treatment alone (Figure 6).

**Figure 7:**
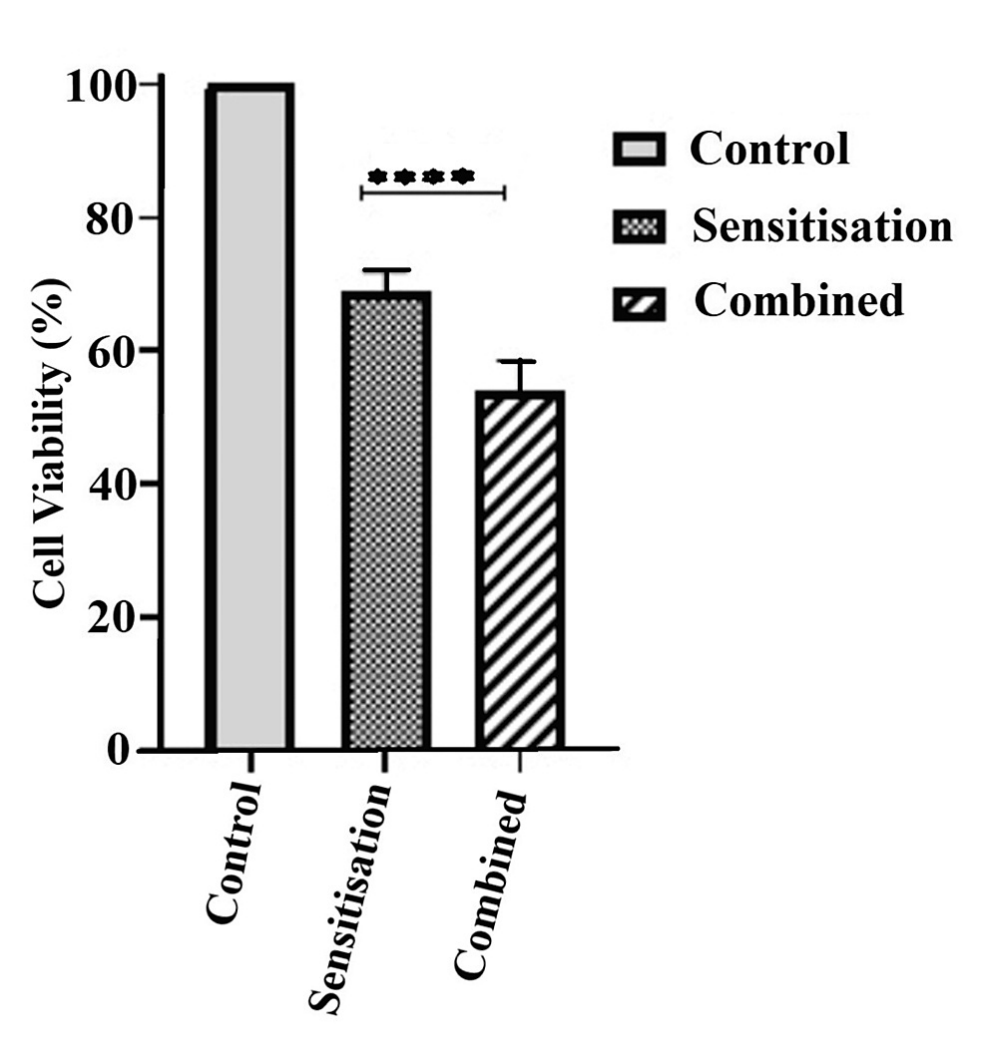
Cytotoxic effect of sensitisation treatment compared to combined treatment The graph shows the cell viability (%) of the KB cells being sensitised with nanocurcumin prior to 24 hours of cetuximab treatment against their combination treatment. Cell viability was higher in the sensitised group compared to the combination treatment group (p<0.0001).

Prior sensitization with NC treatment had less cytotoxic effect than the combination therapy (Figure 7).

## Discussion

We studied the effect of sequential doses of cetuximab and NC treatment individually on oral cancer cells for 24 hours. Oral cancer cells underwent a concentration-dependent cell death with both NC and cetuximab. Cell death by NC treatment (25.4%) was significant (p <0.001) against cetuximab treatment (15.5%). We observed highly significant cell death (p <0.0001) in the NC and cetuximab combined treatment group (46.0%) as compared to each NC (25.4%) and cetuximab (15.5%) monotherapy. The group treated with cetuximab post-NC sensitization (30.9%) showed more significant cell death (p <0.01) than the group with only cetuximab treatment (15.5%).

Our results with cetuximab treatment follow the study findings by Park et al., where significant cytotoxic effects of cetuximab on KB cells were observed at doses starting from 200µg/mL [13]. Cetuximab, a recombinant human-murine chimeric monoclonal antibody, binds explicitly to EGFR on the cell surface, internalizes it, and blocks the downstream signal transduction leading to inhibition of tumor cell proliferation, invasion, metastasis, angiogenesis, and promoting tumor cell apoptosis. The KB cells are known to overexpress the EGFR receptor, which explains the cytotoxic effect seen with cetuximab treatment [14].

The results of NC treatment in our study are similar to the observations made by Srivastava et al., where NC (~200nm size) used in oral cancer cells showed dose-dependent cytotoxicity [15]. Our findings corroborate with the results observed in another study using nanoparticle curcumin (in the range of 34-359.4nm) by Adahoun et al. on prostate cancer cells, where overnight treatment showed a significant cytotoxic effect [16]. In recent years various types of NC in oral cancer cell lines have been studied, and their results were promising cancer cell growth inhibitory and cytotoxic effects [17,18]. A study by Wichitnithad et al. on oral cavity cancer cell line (KB cells) using mono-PEGylated curcumin conjugates (mPEG2000-succinyl-Curcumin, mPEG2000-glutaryl-Curcumin, and mPEG2000- methylcarboxyl-Curcumin) showed cytotoxic results with IC_50_ in the range of 1-3µM, a much lower dose range than our study findings [19].

We observed that the combined treatment with NC and cetuximab was more cytotoxic than the single treatment of each agent, which signifies the adjuvant effect of NC. Duarte et al., in their study, treated two head and neck squamous cell carcinoma (HNSCC) cell lines with the combination of a liposomal form of nanocurcumin and the anticancer drug cisplatin [20]. Our findings are similar to their observation of the combination treatment being more cytotoxic than cisplatin monotherapy. We also found NC treatment to cause more cell death than cetuximab treatment, and a similar effect is observed by Manohar et al. and Chen et al. [21,22]. In our study, the NC-sensitized cetuximab-treated group had highly significant cell death against the only-cetuximab-treated group, highlighting the chemo-sensitizing effect of NC. Similar studies using curcumin molecules on different cancer cell lines support our findings [23,24].

Curcumin has been researched to exert its actions through various pathways exhibiting its anticancer properties as a therapeutic agent. A study by Zhen et al. showed that curcumin inhibited the proliferation of oral cancer cells in a dose-dependent manner along with inhibition of cancer cell invasion and inhibition of activation of both EGFR and EGFR downstream signalling molecules PKB, mitogen-activated protein kinase 1 (MAPK1/2), and signal transducer and activator of transcription 3 (STAT3) [7]. The HNSCC in vitro study models show that curcumin suppresses the activation of transcription factor nuclear factor-kappa B (NF-κB) via the inactivation of inhibitor of nuclear factor-κB (IκKB) activity. The NF-κB inactivation leads to the suppression of many NF-κB-regulated genes involved in cancer development like tumor necrosis factor α (TNF-α), cyclin D1, cellular myelocytomatosis oncogene (c-myc), matrix metalloproteinases (MMP-9), cyclooxygenases (COX-2), and various other interleukins (IL-6,8) [25]. Curcumin has been shown to induce cancer cell autophagy by inhibiting the PKB/mammalian target of rapamycin (mTOR)/p70S6 pathway and MAPK1/2 pathway and inhibits signalling protein STAT3 by suppressing the IL6-mediated phosphorylation of STAT3 [25]. The cytosine-cytosine-adenosine-adenosine-thymidine (CCAAT)/enhancer-binding protein α and insulin-like growth factor binding protein-5, known suppressors of head and neck cancers, are upregulated by curcumin by activating p38 which leads to suppression of oral carcinogenesis [26]. In addition, curcumin modulates the cell cycle by downregulating anti-apoptotic proteins such as B-cell lymphoma 2 (Bcl-2), B-cell lymphoma-extra large (Bcl-xL) and upregulating the apoptotic ones (p53, Bax, Bad, Bim ) causing cell cycle arrest at the G2/M phase [25].

Apoptosis induction, inhibition of cell cycle, expression of anti-apoptotic proteins and angiogenesis, blocking of multiple cell survival signalling pathways, modulation of immune responses, induction of p53 dependent and p53 independent G2/M phase cell cycle arrest are the effects of curcumin. Together, all these factors are likely to be the reason for higher cancer cell death observed with NC treatment than with cetuximab monotherapy (EGFR inhibitor). We also found NC sensitized group to have more cell death than the combined treatment group. Our findings conflicted with the study by Yallapu et al., where pre-treatment of curcumin caused more cell death than the combined treatment (curcumin and cisplatin) [23]. The reason for such observation is to be explored by further studies, including the effect of NC treatment on the downstream molecular pathways, which is a limitation of this study. Future experiments targetting chemotherapeutic dose-lowering effects would further strengthen the outcomes of this study.

## Conclusions

In this lab-controlled cell culture study, we observed the concentration-dependent cytotoxic effect of the NC on the oral squamous cancer cells. There was significant cancer cell death by NC treatment compared to cetuximab monotherapy. The combination treatment had a more substantial effect than single-drug treatment. On sensitization with NC, cetuximab caused more cell death than only cetuximab. All these findings indicate the potential chemo-adjuvant impact of NC. Further studies are needed to determine the molecular mechanisms of NC and its effect on other cancer types with future scope in clinical application.
